# Comprehensive Impact of Multiplanar Malalignment on Prosthetic Mechanics Under Gait Loading After Total Knee Arthroplasty—A Finite Element Analysis

**DOI:** 10.1111/os.70068

**Published:** 2025-05-27

**Authors:** Yichao Luan, Min Zhang, Xiang Dong, Hongping Duan, Zhiwei Wang, Zhichang Li, Cheng‐Kung Cheng

**Affiliations:** ^1^ School of Biological Science and Medical Engineering Beihang University Beijing China; ^2^ Beijing Naton Medical Research Institute Co., Ltd Beijing China; ^3^ Beijing Medical Implant Engineering Research Center Beijing Naton Technology Group Co., Ltd Beijing China; ^4^ Department of Orthopaedics Beijing Chaoyang Hospital, Capital Medical University Beijing China; ^5^ Arthritis Clinical and Research Center Peking University People's Hospital Beijing China; ^6^ School of Biomedical Engineering Shanghai Jiao Tong University, Engineering Research Center of Digital Medicine, Ministry of Education Shanghai China

**Keywords:** finite element analysis, gait loading, multiplanar malalignment, prosthetic mechanics, total knee arthroplasty

## Abstract

**Background:**

Component alignment is a key factor influencing clinical outcomes after total knee arthroplasty (TKA). Previous studies have shown that single‐plane alignment can significantly affect knee joint kinematics and biomechanics. However, the comprehensive impact of multiplanar malalignment has been rarely investigated.

**Objective:**

This study aimed to investigate the influence of the multiplanar malalignment combination on the polyethylene tibial liners under gait loading, a primary activity of daily life, as well as the degree of the influence of the alignments on the different planes.

**Method:**

A validated finite element model of a cruciate‐retaining knee prosthesis under gait loading was used in this study. Five alignment parameters (−5°, −3°, 0°, 3°, 5°) on each plane (coronal, sagittal, and transverse) were selected to simulate clinical alignment errors, resulting in 125 models combining various alignment errors across the three planes. Boundary and loading conditions were set according to ISO 14243‐3:2014. The maximum von Mises stress and contact stress during a gait cycle were recorded for statistical analysis. A polynomial model was used for regression analysis, with the degree of influence of each alignment error on von Mises and contact stress determined by examining the quadratic coefficients.

**Results:**

The highest Mises and contact stress values occurred with alignment errors of 5° varus, 5° flexion, and 5° internal rotation on the coronal, sagittal, and transverse planes, respectively. The lowest stress values were observed with a combination of 3° valgus, 5° flexion, and 0° internal rotation. The regression analysis yielded an *R* ^2^ value of 0.69 between alignment errors and Mises stress, with quadratic coefficients of 0.096, 0.013, and 0.064 for the coronal, sagittal, and transverse alignments, respectively. For contact stress, the *R* ^2^ was 0.697, with quadratic coefficients of 0.083, 0.002, and 0.026 for the coronal, sagittal, and transverse alignments, respectively.

**Conclusion:**

The coronal alignment of the lower limb has the most significant impact on both Mises stress and contact stress of the tibial liner, followed by the rotational alignment of the tibial component. In contrast, the sagittal alignment of the femoral component has the least influence.

## Graphical abstract

The mechanics of tibial liners with various multiplanar malalignment combinations on the coronal, sagittal, and transverse planes under gait loading were analyzed. Results indicated that coronal alignment of the lower limb had the most significant impact on both Mises stress and contact stress in the tibial liner, followed by the rotational alignment of the tibial component. In contrast, sagittal alignment of the femoral component had the least influence.

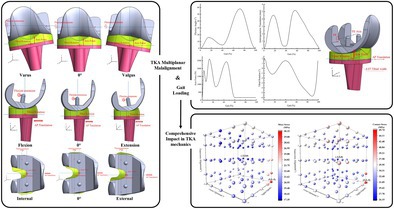

## 1 Introduction

Total knee arthroplasty (TKA) is widely regarded as one of the most effective treatments for severe osteoarthritis of the knee, demonstrating acceptable clinical outcomes over the past decades [1, 2, 3]. Despite significant advancements in prosthesis materials, design, surgical techniques, and tools, some patients remain dissatisfied with the outcomes after TKA [4, 5]. Revision TKA due to aseptic loosening, restricted range of motion, anterior pain, and other complications was reported frequently [6, 7, 8]. According to previous studies, malalignment has been one of the major factors that lead to dissatisfaction and revision. 13% of revisions resulting from malalignment were collected from the Dutch Arthroplasty register in 2023 [9], and 21% of patients underwent the revised procedures because of malalignment in Thiele's study [10]. Tibial malrotation, in particular, has been identified as a risk factor for knee pain and suboptimal functional outcomes following TKA [11].

The alignment of prosthetic components critically influences knee joint kinematics and biomechanics, which are essential for favorable clinical outcomes and prosthesis longevity [12, 13, 14, 15]. Fang et al. demonstrated that tibial rotational alignment impacts the anterior–posterior translation of the femoral component, with moderate external rotation of the tibial component enhancing postoperative kinematics [16]. Suh et al. investigated the influence of the coronal alignment on the knee joint; the results showed that varus alignment resulted in higher contact stress of the femorotibial joint than valgus [17]. Furthermore, Koh et al. reported that different sagittal alignments of the femoral component induce distinct knee kinematic and biomechanical changes [18]. However, most studies focus solely on alignment errors within a single plane, overlooking the combined effects of misalignments across multiple planes.

Emerging evidence highlights the interplay between alignments on different planes. Betzle et al. observed that varying the tibial slope from 0° to 7° altered the coronal plane alignment from 0° to 2.4° varus when conventional instrumentation was used, with less variability when using navigation systems [19]. D'Lima reported that tibial rotation significantly affects coronal alignment: a 25° internal tibial rotation produced a 3° varus alignment, while a 15° external rotation shifted alignment to 2° valgus [20]. These findings underscore the simultaneous existence and interdependence of alignment errors across different planes.

The purposes of this study are as follows: (i) to investigate the contributions of alignment errors in different planes to prosthetic mechanics; (ii) to analyze the combined influence of malalignment across multiple planes on prosthetic mechanics.

## 2 Materials and Method

### 2.1 Finite Element Model of Knee Prosthesis

This study used a validated cruciate‐retaining prosthesis model (Size D, NexGen CR‐Flex, Zimmer Inc., Warsaw, IN, United States), comprising the femoral component, tibial liner, and bearing [21]. The elastic modulus and Poisson's ratio for the femoral and tibial components were 220 GPa and 0.3, respectively. The tibial liner was modeled as ultrahigh molecular weight polyethylene, with an elastic modulus of 495 MPa and a Poisson's ratio of 0.4 [22].

**FIGURE 1:**
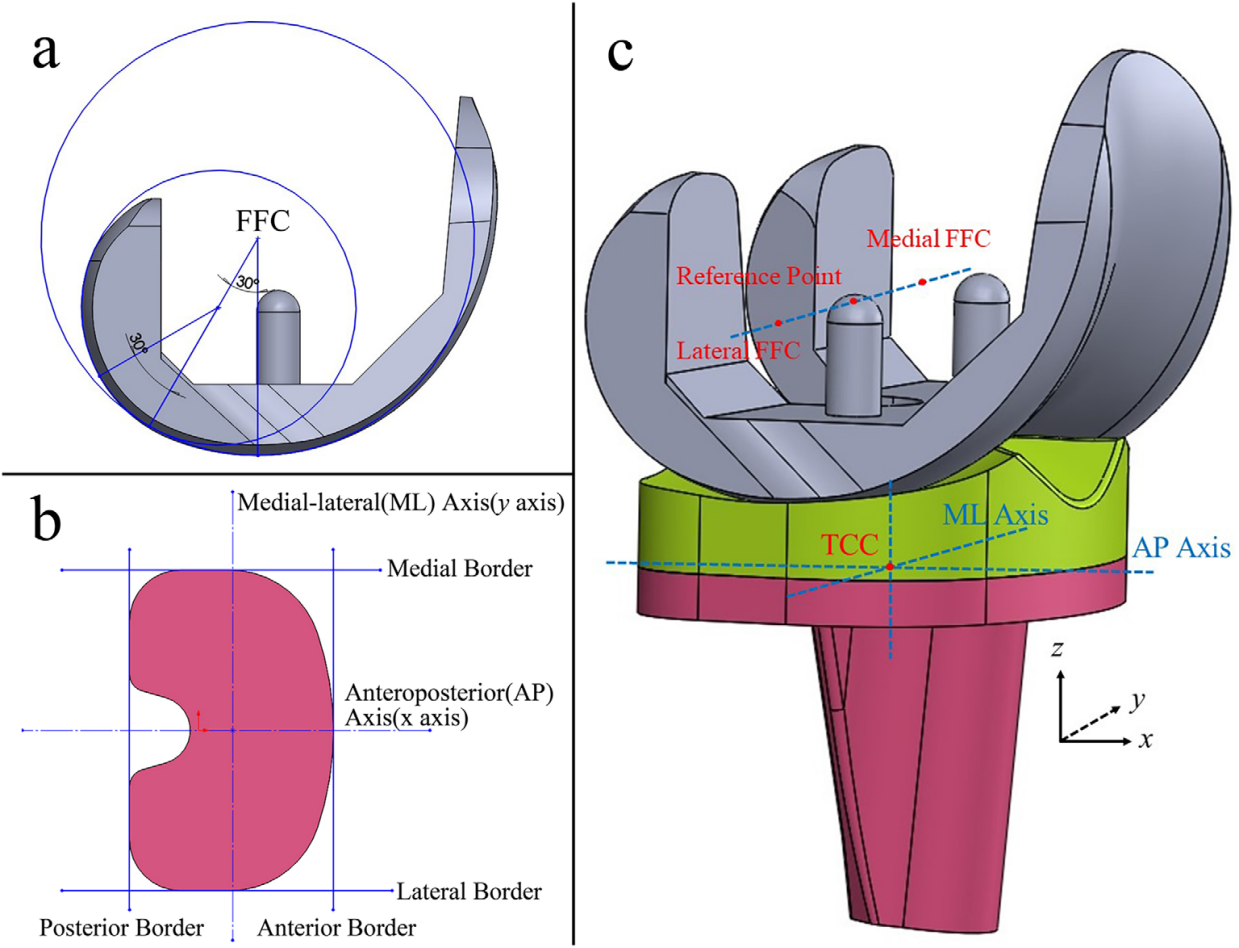
3D model of the knee prosthesis (a) definition of the femoral flexion center (FFC); (b) anteroposterior and medial‐lateral axes of the tibial component; (c) the coordinate system of the model, TCC represents tibial component center.

The model's coordinate system was established based on ISO 14243‐3:2014. For the femoral component, the medial and lateral femoral flexion centers (FFCs) were defined as the intersection points of the normals to the contact points when the femoral component was flexed to 30° and 60°. The midpoint between the medial and lateral FFCs served as the reference point for the femoral component. The flexion‐extension (FE) axis was defined as the line connecting the medial and lateral FFCs (Figure 1a). In the coordinate system for the tibial component, the anteroposterior (AP) axis (*x*‐axis) was defined as the central line between the most lateral and medial borders. A line perpendicular to the AP axis established the medial‐lateral (ML) axis (*y*‐axis) (Figure 1b), with the intersection of the AP and ML axes marking the tibial component center (TCC) on the upper surface. The *z*‐axis was then defined as a line perpendicular to the upper surface, passing through the TCC (Figure 1c).

The femoral and tibial components were modeled as rigid bodies, while the liner was set as deformable. Assembly was achieved by aligning the most distal points of the femoral condyles with the lowest points of the polyethylene tibial liner. The liner was bonded to the tibial component, and the femoral component was set to contact the tibial liner with a friction coefficient of 0.04 [23].

### 2.2 Boundary and Loading Conditions

**FIGURE 2:**
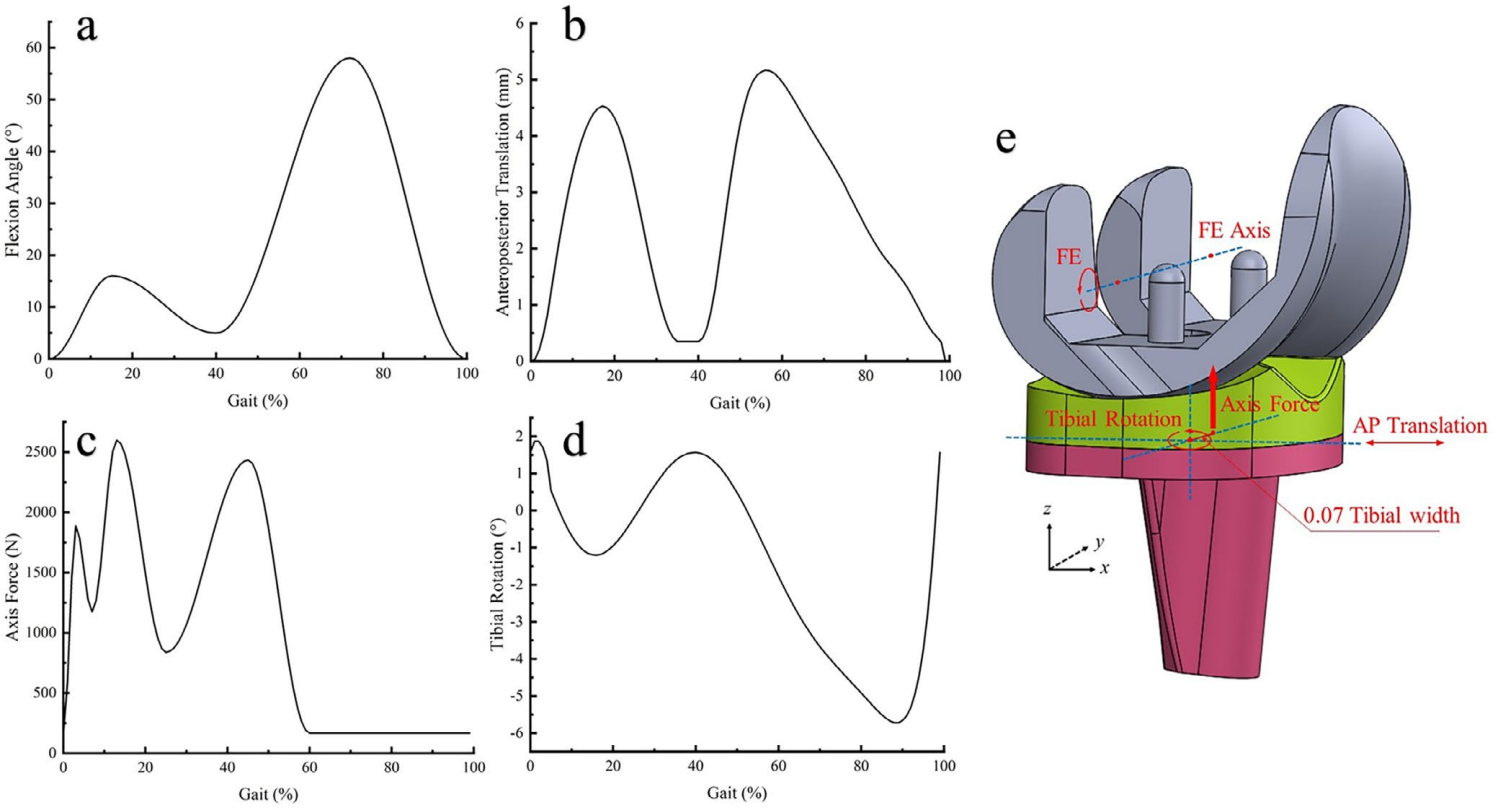
Input curves and loading conditions (a: flexion angle; b: anteroposterior translation, c: axial force, d: tibial rotation, e: schematic diagram of loading conditions).

The femoral component was constrained to flex solely around the flexion‐extension axis, while the tibial component was restricted from flexion‐extension movement but allowed to move freely in all other degrees of freedom [15, 16, 21]. Dynamic simulations of a complete gait cycle were conducted in Abaqus 2017 (Dassault Systèmes Simulia Inc., France), with each cycle lasting 1 s, as specified by ISO 14243‐3:2014. Four curves were loaded into the model to simulate the gait cycle, including flexion‐extension, anteroposterior translation, axial force, and tibial rotation (Figure 2a–d). Flexion‐extension was applied at the femoral reference point around the FE axis, while the axial force was applied along the *z*‐axis, offset medially by 0.07 times the tibial component width. Both the AP translation and tibial rotation were centered on the tibial component (Figure 2e).

### 2.3 Simulation of Different Malalignment Errors

**FIGURE 3:**
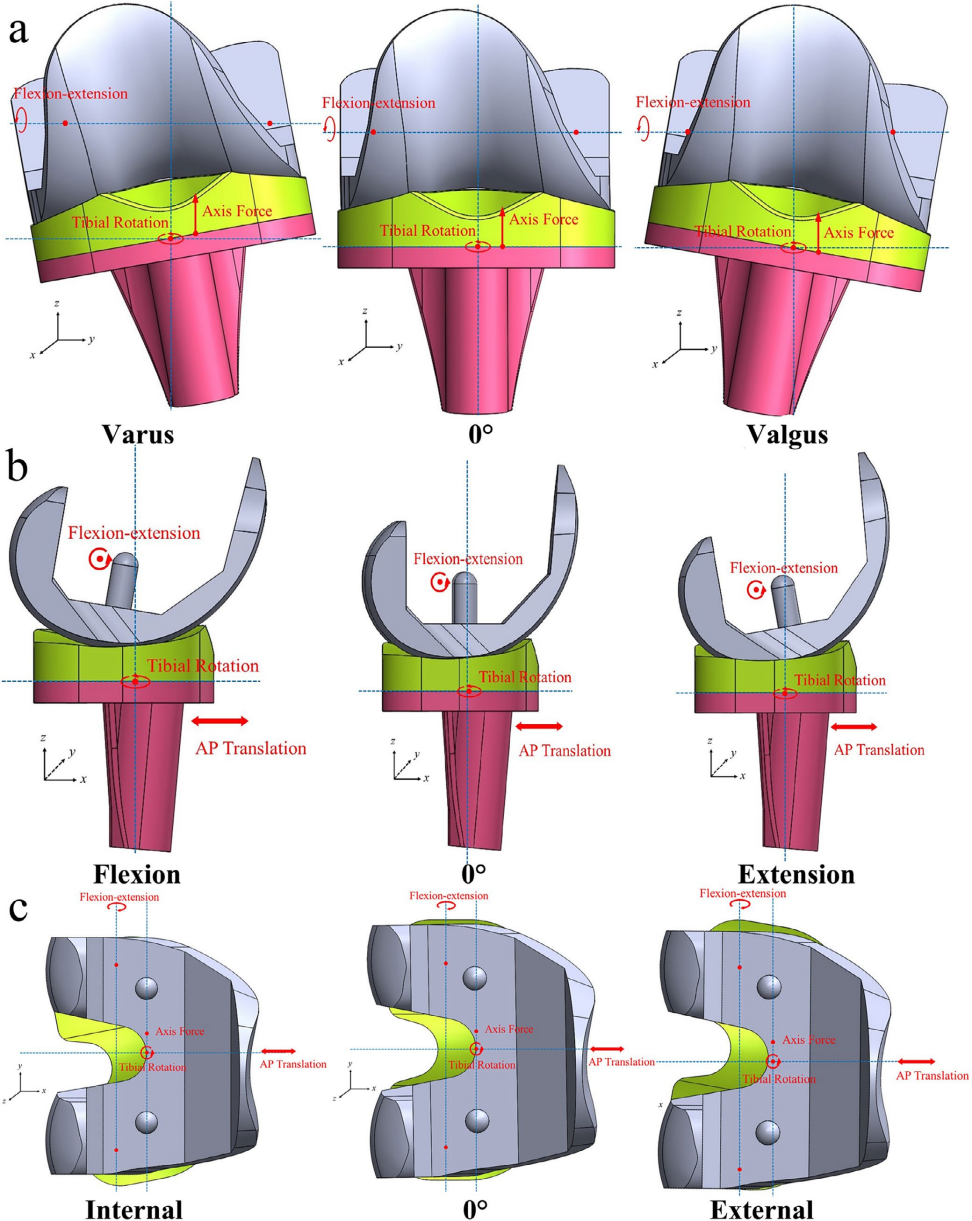
Malalignment of the knee prosthesis (a: coronal plane; b: sagittal plane; c: transverse plane).

Three key alignment aspects of the knee joint prosthesis were analyzed in this study: varus‐valgus alignment of the lower limb on the coronal plane, flexion‐extension of the femoral component on the sagittal plane, and internal–external rotation of the tibial component on the transverse plane. To simulate varus‐valgus malalignment, the knee prosthesis was rotated around the AP axis. Flexion‐extension adjustments were applied to the femoral component along the FE axis. Additionally, rotational malalignment was introduced by rotating the tibial component internally or externally around the *z*‐axis during model assembly (Figure 3). Alignment deviations of −5°, −3°, 0°, 3°, and 5° were applied in each plane, with these values then randomly combined to create various alignment conditions. A total of 125 unique alignment combinations were generated and modeled for analysis under gait loading refer to ISO 14243‐3:2014. The maximum von Mises stress and contact stress of the tibial liner under the gait loading were presented for analysis.

### 2.4 Statistical Analysis

The relationship between tibial liner mechanics and alignment errors is generally considered to follow a parabolic trend based on the previous studies [21]. Therefore, the polynomial model was selected for the regression analysis of the results. The degree of influence of each alignment error on the Mises and contact stress was assessed by the quadratic coefficients. The polynomial model is represented as follows:

$$y=a+bx_{1}+cx_{2}+dx_{3}+ex_{1}^{2}+fx_{2}^{2}+gx_{3}^{2}+hx_{1}x_{2}+ix_{2}x_{3}+jx_{1}x_{3}$$

In the model, *y* represents either the Mises stress or contact stress, while *x*_1_, *x*_2_, and *x*_3_ represent coronal, sagittal, and transverse alignment errors, respectively. The terms *a*, *b*, *c*, *d*, *e*, *f*, *g*, *h*, *i*, and *j* are constants in the polynomial equation.

Using 125 sets of simulation results, the least squares method was applied to derive regression parameters with minimal error. All statistical analyses were conducted using SPSS 26.0 (IBM Inc., Chicago, USA).

## 3 Results

### 3.1 Nonlinear Regression Analysis of Malalignment Errors and Tibial Liner Stress

The maximum Mises stress and contact stress for various alignment combinations under gait loading were extracted. Table 1 presents the multivariate nonlinear regression results, showing the relationship between alignment errors across different planes and the maximum Mises stress on the liner. The regression model achieved an *R* ^2^ value of 0.690, with the regression equation as follows:

$$\begin{array}{c} y_{\text{mises stress}}=18.961+0.176x_{1}-0.047x_{2}-0.135x_{3}+0.096x_{1}^{2}+0.013x_{2}^{2}\\ \;+0.064x_{3}^{2}+0.038x_{1}x_{2}-0.049x_{2}x_{3}+0.027x_{1}x_{3} \end{array}$$

**TABLE 1:** Regression parameters of alignment error and Mises stress.

Parameters	Estimated value	Standard deviation	95% confidence interval	
			Lower	Upper
*a*	18.961	0.274	18.419	19.503
*b*	0.176	0.029	0.119	0.233
*c*	−0.047	0.029	−0.104	0.010
*d*	−0.135	0.029	−0.191	−0.078
*e*	0.096	0.011	0.075	0.118
*f*	0.013	0.011	−0.009	0.034
*g*	0.064	0.011	0.043	0.085
*h*	0.038	0.008	0.023	0.053
*i*	−0.049	0.008	−0.064	−0.033
*j*	0.027	0.008	0.011	0.042

### 3.2 Nonlinear Regression Analysis of Malalignment Errors and Tibial Liner Pressure

The estimated quadratic coefficients for alignment errors in the coronal, sagittal, and transverse planes were 0.096 (SD: 0.011, 95% CI: 0.078–0.118), 0.013 (SD: 0.011, 95% CI: −0.009–0.034), and 0.064 (SD: 0.011, 95% CI: 0.023–0.053), respectively. The regression analysis indicated that the coronal alignment errors had the greatest impact on maximum Mises stress under gait loading, followed by the transverse alignment error, with the sagittal alignment error exerting the least influence.

The regression result of alignment errors and contact stress of the tibial liner is shown in Table 2. The estimated quadratic coefficients for alignment errors in the coronal, sagittal, and transverse planes were 0.083(SD:0.017, 95% CI:0.049–0.116), 0.002(SD:0.017, 95% CI:−0.032 to 0.036)and 0.064(SD:0.017, 95% CI:−0.007 to 0.060), respectively. The *R* ^2^ of the regression is 0.697, and the equation is shown as follows. The results align with those observed for Mises stress, where coronal alignment has the greatest influence on contact stress, while sagittal alignment exhibits the lowest impact.

$$\begin{array}{c} y_{\text{Contact Stress}}=31.124+0.353x_{1}-0.514x_{2}-0.065x_{3}+0.083x_{1}^{2}+0.002x_{2}^{2}\\ \;+0.026x_{3}^{2}+0.043x_{1}x_{2}-0.069x_{2}x_{3}+0.028x_{1}x_{3} \end{array}$$

**TABLE 2:** Regression parameters of alignment error and contact stress.

Parameters	Estimated value	Standard deviation	95% confidence interval	
			Lower	Upper
*a*	31.124	0.434	30.265	31.984
*b*	0.353	0.045	0.263	0.443
*c*	−0.514	0.045	−0.604	−0.424
*d*	0.065	0.045	−0.025	0.155
*e*	0.083	0.017	0.049	0.116
*f*	0.002	0.017	−0.032	0.036
*g*	0.026	0.017	−0.007	0.060
*h*	0.043	0.012	0.018	0.067
*i*	−0.069	0.012	−0.093	−0.044
*j*	−0.028	0.012	−0.053	−0.004

### 3.3 Combined Influence of Malalignment Across Multiple Planes on Prosthetic Mechanics

**FIGURE 4:**
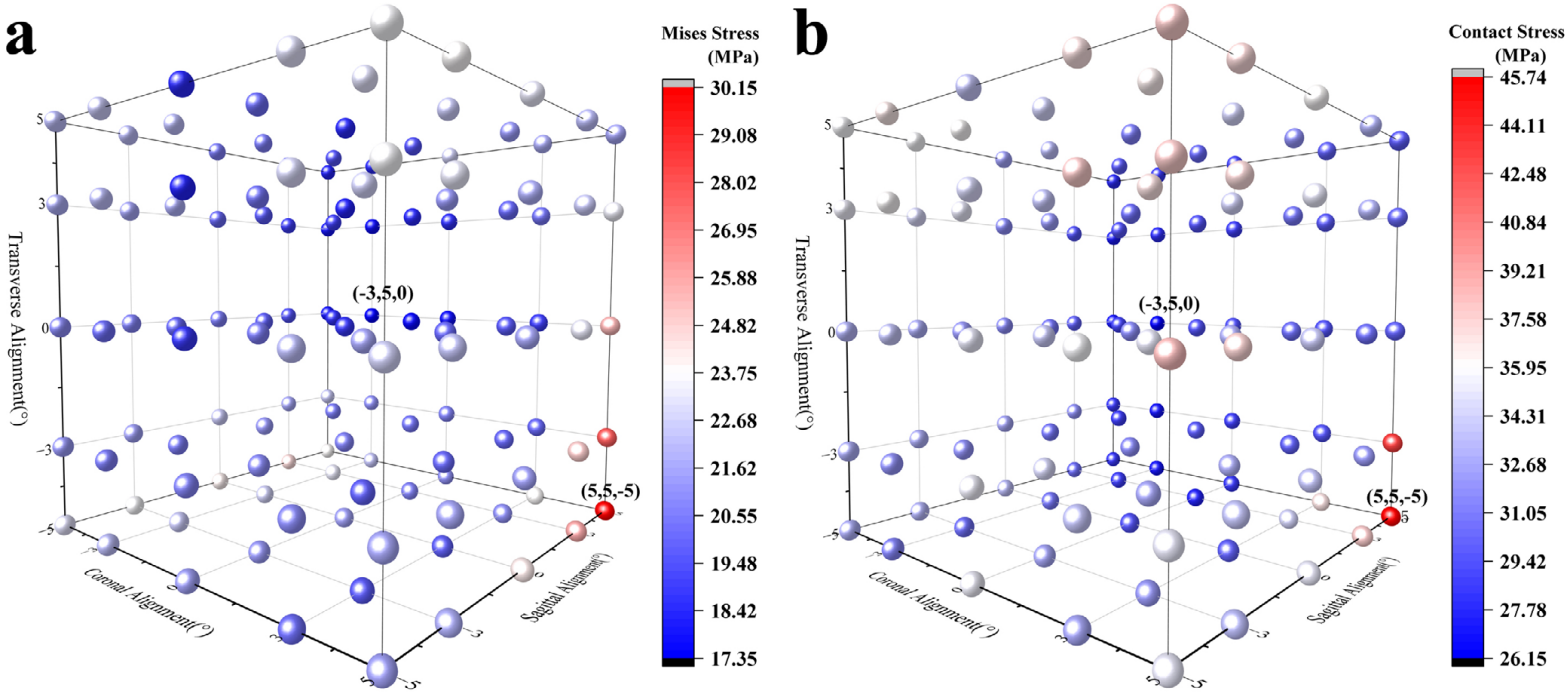
Mechanics of tibial liner under different alignment combinations (a: mises stress, b: contact stress).

Figure 4 illustrates the Mises stress and contact stress on the tibial liner under various alignment combinations. The maximum Mises stress of 30.15 MPa and contact stress of 45.74 MPa occurred when the lower limb alignment was 5° varus, with the femoral component flexed at 5° and the tibial alignment set to 5° internal rotation. The minimum Mises stress, recorded at 17.35 MPa, was observed with a lower limb alignment of −3° valgus, the femoral component flexed at 5°, and the tibial alignment at 0° on the transverse plane, resulting in a contact stress of 26.15 MPa.

## 4 Discussion

The primary finding of this study is that alignment errors across different planes exert varying levels of influence on the mechanics of the tibial liner under gait loading. The results indicate that the coronal alignment of the lower limb has the most significant impact on both Mises stress and contact stress of the tibial liner, followed by the rotational alignment of the tibial component. In contrast, the sagittal alignment of the femoral component has the least influence.

### 4.1 Contribution of the Malignment Errors of Different Planes

Component alignment has a significant impact on postoperative outcomes. In clinical practice, it is generally recommended that lower limb alignment errors in the coronal plane do not exceed 3°. In the sagittal plane, femoral component flexion should be limited to within 5°, with hyperextension being avoided. The tibial component is typically positioned with a posterior slope of 3°–7°. For rotational alignment, the femoral component is ideally aligned parallel to the transepicondylar axis, with a recommended error margin of less than 3°. Similarly, the tibial component is aligned with reference to the Akagi line, also within a 3° error range.

Coronal alignment has the most significant impact on the mechanics of the knee joint, directly influencing the survival of knee prostheses. Fang reported that the survival rate of total knee arthroplasties (TKAs) was highest when the coronal alignment between the femoral and tibial anatomical axes was within 3°–7°, based on a follow‐up study of 6070 TKAs [14]. Similarly, Ritter emphasized the importance of achieving neutrality in all three alignments to maximize implant survival [24]. Therefore, ensuring coronal alignment falls within the neutral range during surgery is critical for TKA longevity. Recently, advancements in surgical tools have significantly improved the accuracy of TKA procedures. Technologies such as navigation systems, patient‐specific instrumentation, and robotic systems have enhanced the precision of surgical alignment, particularly benefiting younger and less experienced surgeons [25, 26, 27]. Additionally, coronal alignment tolerance should be considered when designing knee prostheses. Features such as rotating platforms, lower conformity, and inherent varus‐valgus characteristics hold potential for enhancing tolerance to coronal malalignment.

In this study, gait cycle loading conditions were based on ISO 14243‐3:2014, an in vitro wear test standard for knee prostheses. The maximum axial force of 2600 N was applied perpendicular to the tibial component's bearing surface when the prosthesis was in the standard position. Coronal alignment errors of the lower limb inclined the tibial bearing surface, leading to variations in the axial force's loading direction. Sagittal alignment of the femoral component and rotational alignment of the tibial component impacted the liner's mechanics through changes in contact area due to shifts in the initial contact position of the femoral‐tibial joint. The flexed femoral component caused the contact point on the tibial liner to move slightly anteriorly during gait loading, where a smaller curvature surface increased contact area, thereby reducing contact pressure and stress. Conversely, extension malalignment shifted the contact posteriorly, raising contact pressure and stress. In this study, both internal and external tibial malrotation on the transverse plane induced higher stress and contact pressure on the tibial liner compared to a neutrally aligned prosthesis. This was likely due to contact position shifts and area changes, as observed with sagittal malalignment. During gait loading, the tibial component rotated internally by nearly 6°, with internal malrotation increasing tibial rotation. The anteroposterior translation of the femoral condyles further led to a more posterior‐lateral contact position due to internal malrotation, reducing contact area and increasing stress. Therefore, femoral flexion‐extension in the sagittal plane and tibial rotation in the transverse plane did not alter the axial force's direction. Besides, sagittal malalignment primarily alters the AP position of the initial contact point, while rotational malalignment changes both the AP and mediolateral (ML) positions of the contact point. Variations in the ML direction result in greater stress concentration due to the larger coronal curvature compared to sagittal curvature. As a result, malalignment on the transverse plane induces higher stress compared to malalignment on the sagittal plane. This sensitivity to loading direction likely explains why coronal alignment errors have the greatest impact on tibial liner mechanics.

According to ISO 14243‐3, femoral anteroposterior loading and tibial rotational loading are displacement‐controlled, making the initial contact position on the articular surface a critical factor that significantly affects mechanics during gait loading. Internal malalignment results in a more anterior contact position on the lateral side. Similarly, flexed malalignment of the femoral component also shifts the contact position anteriorly. The larger curvature and increased thickness on the anterior side of the tibial liner lead to tighter contact and higher stress in these regions.

### 4.2 Influence of the Malalignment of the Low Limb

Increased Mises and contact stress were observed with varus alignment of the lower limb compared to valgus alignment. The axial force loading point was positioned medially at a distance of 0.07 times the width of the tibial component, as specified by ISO 14243‐3:2014, creating an unbalanced mechanical load on the tibial liner. With varus alignment, higher stress is concentrated on the medial side, leading to increased overall liner stress. In contrast, a slight valgus alignment could counterbalance the medial offset of the loading point. Previous studies have highlighted the relationship between contact pressure and polyethylene wear. It has been shown that wear rates of polyethylene are not highly dependent on contact stress below 6.9 MPa, but above this threshold, wear rates increase substantially [28]. In this study, the contact pressures for different malalignment scenarios exceeded 6.9 MPa, suggesting that higher contact pressures contributed to increased wear. Li et al. reported that varus alignment increased polyethylene damage scores on the articular surface compared to valgus alignment, as shown in retrieval analysis, which aligns with the findings of this study [13]. Previous research further supports that the clinical performance of polyethylene liners is linked to mechanical stress, with greater stress associated with more pitting and delamination [29, 30].

### 4.3 Factors Influencing the Study Results

The material properties of the tibial liner could significantly influence the results. Previous studies have shown that the polyethylene composition and manufacturing methods affect the mechanics of tibial liners [31, 32, 33]. High‐crosslinked polyethylene (HXLPE) has demonstrated superior mechanical properties and wear performance compared to conventional polyethylene (CPE) and ultrahigh‐molecular weight polyethylene (UHMWPE). In this study, UHMWPE was selected based on findings from existing literature [22]. However, variations in polyethylene materials may lead to discrepancies in the results. Daily activity variations are often influenced by cultural and regional demands, such as full squatting and cross‐leg sitting in Asia. Common daily activities include gait, stair ascent and descent, squatting, sit‐stand‐sit transitions, and pivot turns. Among these, gait is the most fundamental and representative activity and was the focus of this study. Previous research has investigated the impact of different gait loading conditions on mechanics and wear performance [34, 35]. Standards such as ISO 14243 and ASTM F3141 are typically used to evaluate wear, with the ISO standard being more widely adopted. The choice of gait loading standard may also contribute to variations in study results.

### 4.4 Strengths and Limitations

This study has several strengths. First, the influence of the multiplanar malalignment on the prosthetic mechanics was investigated, in which the malalignment conditions were close to the clinical employment of the knee prosthesis. Besides, the nonlinear regression analysis indicated the contribution of the malalignment of the different planes, which was helpful for the surgical plans.

This study also has several limitations. First, only a cruciate‐retaining prosthesis was analyzed, so the findings may not generalize to other types of knee prostheses, such as posterior‐stabilized (PS) designs. The primary design difference between these prostheses lies in their mechanism for preventing anterior translation of the femoral component: PS prostheses utilize a postcam mechanism, while CR prostheses rely on the posterior cruciate ligament. The presence of the post in the tibial liner of PS prostheses may result in abnormal contact under malalignment conditions, leading to differences in sensitivity to malalignment. Further studies and robust evidence are needed to confirm this assumption. Additionally, only five alignment errors were considered per plane; including a broader range of alignment errors and combinations could enhance the accuracy of the multivariate nonlinear regression model. Furthermore, this study did not investigate the effects of the tibial posterior slope or femoral component malrotation, which should be addressed in future research. Finally, loading conditions were limited to the gait cycle; future studies could consider additional activities, such as stair ascent and descent or squatting, to better capture prosthetic performance.

## 5 Conclusion

The combination of alignment errors across multiple planes significantly impacts the mechanical performance of the tibial liner under gait loading. Among the factors analyzed—varus‐valgus alignment of the lower limb axis, flexion‐extension of the femoral component, and internal‐external rotation of the tibial component—varus‐valgus alignment has the most significant impact on the mechanics of the liner, while the flexion‐extension of the femoral prosthesis has the least effect.

## Author Contributions

**Yichao Luan:** writing – original draft, conceptualization, investigation, methodology, software, data curation, visualization. **Min Zhang:** formal analysis, software, validation, visualization, review and editing. **Xiang Dong:** resources, funding acquisition, investigation, review and editing. **Hongping Duan:** data curation, formal analysis, review and editing. **Zhiwei Wang:** resources, methodology, funding acquisition, review and editing. **Zhichang Li:** conceptualization, methodology, resources, review and editing. **Cheng‐Kung Cheng:** conceptualization, resources, supervision, funding acquisition, review and editing.

## Conflicts of Interest

The authors declare no conflicts of interest.
